# Reaching Distance Influences Perceptual Decisions

**DOI:** 10.1111/ejn.70006

**Published:** 2025-02-02

**Authors:** Eleonora E. Assarioti, Robert J. van Beers, Jeroen B. J. Smeets, Bernadette C. M. van Wijk

**Affiliations:** ^1^ Department of Human Movement Sciences, Faculty of Behavioural and Movement Sciences Vrije Universiteit Amsterdam Amsterdam The Netherlands; ^2^ Donders Institute for Brain, Cognition and Behaviour Radboud University Nijmegen The Netherlands; ^3^ Department of Neurology, Amsterdam University Medical Centers, Amsterdam Neuroscience University of Amsterdam Amsterdam The Netherlands

**Keywords:** action, decision making, embodied choice, motor costs, perception, random dot motion

## Abstract

Decision making is an integral part of everyday life. Recently, there has been a growing interest in the potential influence of action on perceptual decisions, following ideas of embodied decision making. Studies examining decisions regarding the direction of noisy visual motion have found a bias towards the least effortful response option in experiments in which the differences in motor costs associated with alternative response actions were implicit, but not in an experiment in which these differences were made explicit. It remains unclear whether the biasing effect generalizes to other perceptual tasks than motion perception and whether consciously experiencing motor costs prevents such biases. To test the generalizability of effects across perceptual tasks, we used a within‐subjects design where 24 participants performed both a motion discrimination task and an orientation discrimination task. Motor costs were manipulated by presenting response buttons for the two alternative choices at different reaching distances. By varying distances randomly, we avoided implicit biases linked to specific decisions. Our findings revealed a bias towards closer response options in both tasks, indicating that explicit information of motor costs significantly impacts perceptual decisions beyond motion discrimination. Contrary to prevailing theories that consider the motor system as a mere effector of the decision, our study implies that the actions that are associated with the response options influence the decision process itself.

## Graphical abstract

Participants performed two perceptual discrimination tasks in which we manipulated the motor costs of the two response options by varying the distance to the response buttons. We found a bias towards choosing closer options in both tasks, indicating that explicit motor costs influence perceptual decisions. This challenges the view that the motor system merely executes decisions.

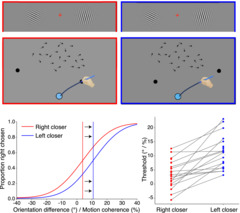

**Abbreviations**
M: mean
Mdn: median
SD: standard deviation

## 1 Introduction

From selecting what to wear in the morning to choosing a career path, the decisions we make as we navigate the world around us are integral to shaping our everyday experiences and interactions. The impact of altered daily‐life decision making becomes starkly evident in various mental disorders and neurodegenerative diseases such as substance use, mood and anxiety disorders, schizophrenia, Alzheimer's disease and frontotemporal dementia, where individuals struggle with simple choices or show risk‐seeking behaviour (Gleichgerrcht et al. 2010; Paulus 2007). Consequently, extensive research has focused on understanding the neural basis of decision making, defined as the cognitive processes involved in reaching a decision and the various contextual factors that influence this process (Bland and Schaefer 2012; Gold and Shadlen 2007; Rangel, Camerer, and Montague 2008). Decision making encompasses various levels of cognitive complexity, with perceptual decision making being perhaps the most straightforward to study. Therefore, researchers have shown particular interest in this area, primarily focusing on the features of stimuli that influence choices, on the effect of stimulus expectation and task instructions such as a focus on speed versus accuracy and on disentangling the role of different brain regions (e.g., Bogacz et al. 2010; Gold and Shadlen 2007; Heekeren, Marrett, and Ungerleider 2008; Summerfield and de Lange 2014).

Most research paradigms in the field of perceptual decision making require participants to make choices based on sensory information and to report them through button presses, with choices and reaction times serving as primary outcome measures. These studies align with the prevailing theory of serial processing in decision making, which suggests that the process starts with the acquisition of information from the environment and concludes with the execution of a decision through a motor command (Donders 1969; Gold and Shadlen 2007; Oppenheimer and Kelso 2015). Recently, however, there has been a growing interest in the role of the motor system itself in perceptual decision making (Carsten, Fievez, and Duque 2023; Connors and Rende 2018; Grießbach et al. 2022; Lepora and Pezzulo 2015; Wispinski, Gallivan, and Chapman 2018). Specifically, it has been argued that the decisions we make are constrained and steered by our ability and ease to physically interact with the environment (Cisek and Kalaska 2010). For example, biomechanical properties of arm reaching movements influence action selection with a bias towards the least effortful movement, as evidenced in both free choice scenarios (Bakker et al. 2017; Cos, Bélanger, and Cisek 2011; Schweighofer et al. 2015) and goal‐related paradigms, where participants were instructed to tap on as many targets as possible (Brenner and Smeets 2015, 2022). Hence, in the emerging field of *embodied decision making*, action is not merely viewed as an output channel. Instead, a bidirectional influence between perceptual, cognitive and motor systems is argued to determine our decision‐making behaviour (Cisek 2012).

Several studies have explored the role of action in perceptual decision making by focusing on the influence of motor costs on reporting perceptual judgements. Such influence might arise due to an asymmetry in the motor responses associated with alternative response options. Marcos et al. (2015) found that if participants have to report perceptual choices with arm movements that differ in their intrinsic biomechanical costs due to their movement trajectory, the perceptual decisions are biased towards the option requiring the least effort, even if negatively impacting performance. Furthermore, Hagura, Haggard, and Diedrichsen (2017) showed that perceptual decisions are influenced by the physical resistance associated with executing the motor response. In their experiment, progressive resistance was applied to one of two possible motor responses, with participants being unaware of this manipulation. The study showed that after repeated exposure to the resistance, there was a bias towards the less effortful option. This bias persisted even when participants only had to verbally report their choices, which led the researchers to argue that the influence of motor costs fundamentally altered how sensory inputs are transformed into decisions. However, these findings are not conclusive when it comes to the generalization of the effect to other decision‐making contexts and aspects of motor costs.

In particular, both Marcos et al. (2015) and Hagura, Haggard, and Diedrichsen (2017) employed an implicit manipulation of motor costs, while in real life, people are frequently aware of the effort of their movements. This prompted Manzone and Welsh (2023) to examine the influence of explicit motor costs on decision making. In their experiment, the effort to move was manipulated by means of attaching a resistance band to the participant's hand, thus requiring more force to move in one direction versus the opposite direction. According to their findings, decision making was not influenced by explicit movement effort, which led the authors to argue for a context‐specific influence of motor costs on perceptual judgements. Nevertheless, only one explicit manipulation of motor costs (i.e., force) was tested, and it remains unclear whether other forms of explicit motor cost manipulations, such as reaching distance, equally show a lack of influence on perceptual decision making. Moreover, all three studies relied on the same perceptual task: the random dot motion task in which participants decide on the perceived left–right direction of motion of a cloud of dots. Although this is a well‐studied and modelled perceptual discrimination task (e.g., Forstmann, Ratcliff, and Wagenmakers 2016), it is possible that the dynamic visual characteristics of the stimulus (leftward or rightward motion) might prompt the corresponding movement, thus influencing action selection. Therefore, it remains unclear to what extent the influence of motor costs on perceptual decision making generalizes to other types of perceptual tasks, such as orientation discrimination, for which the stimulus dimension does not prompt a response.

In the present study, we investigated whether reaching distance influences perceptual decision making in two perceptual tasks: a motion discrimination task, using the random dot motion task, and an orientation discrimination task using two static Gabor wavelet stimuli. Orientation discrimination tasks using Gabor stimuli have often been used in the field of perceptual decision making (e.g., Balsdon, Wyart, and Mamassian 2020; McGovern, Webb, and Peirce 2012; Nuiten et al. 2023). Response buttons for left and right choice options were presented at different reaching distances on a touchscreen. We hypothesized that a shorter reaching distance would introduce a bias towards the corresponding choice. Our manipulation, although not subtle, was also not predictable since the least effortful option appeared with the same probability for both directions and was randomly determined between trials; hence, participants could not associate one direction with the least effortful movement. Our findings revealed that reaching distance indeed biases response choices in both experimental tasks. Hence, our study provides evidence that explicit motor costs in the form of reaching distance can influence perceptual judgements in a systematic way across perceptual tasks.

## 2 Methods

### 2.1 Participants

A predetermined sample size of 24 participants—university students participating in exchange for course credits—were recruited for the present study. This sample size allowed for an exact counterbalanced order in which participants performed the two experimental tasks and the two blocks within each task. Previous studies examining the effect of action on perceptual decision making recruited fewer participants (Hagura, Haggard, and Diedrichsen 2017; Manzone and Welsh 2023; Marcos et al. 2015). However, to be able to detect potentially smaller effects for a different perceptual task and motor cost manipulation, we opted for a bigger sample. Assuming a statistical power of 80%, the smallest effect size that can be reliably detected for this sample size in a two‐tailed one sample *t*‐test or paired samples *t*‐test with significance level of 0.05 equals *d*/*d*_*z*_ = 0.60 (G*Power sensitivity analysis, Faul et al. 2007). All participants reported normal or corrected‐to‐normal vision and no motor impairments of the arm. We excluded two participants after data collection for different reasons. One participant reported problems with peripheral vision during the experiment and was excluded from both tasks. We also carefully inspected all psychometric curves for which the lapse rate was estimated to be more than 0.05 (5%) (see Section 2.3). This led to the exclusion of one participant from the motion discrimination task. Therefore, the final sample size for the analyses regarding the motion discrimination task consisted of 22 individuals (five males) with reported ages of 18–31 years (M = 21.2, SD = 3.1), while the final sample size for the orientation discrimination task consisted of 23 individuals (six males) with reported ages of 18–31 years (M = 21.1, SD = 2.0). The study was approved by the Scientific and Ethical Review Board of the Faculty of Behavioural and Movement Sciences.

### 2.2 Experimental Design

The experiment consisted of two tasks that comprised different stimulus categories: a motion discrimination task with random dot motion stimuli and an orientation discrimination task with Gabor wavelet stimuli. During the motion discrimination task, participants were presented with a cloud of moving dots. A percentage of dots moved coherently towards the left or towards the right, while the remaining dots moved in random directions. Participants reported the perceived overall right‐ or leftward movement. During the orientation discrimination task, participants were presented with two Gabor wavelets and reported whether the left or right one was oriented more towards the vertical. Stimulus presentation and response recording were done via a touchscreen on which participants slid a stylus towards one of the two displayed response buttons to indicate their response. The response buttons were presented asymmetrically, in such a way that either the left button was presented at a short reaching distance and the right button at a long reaching distance, or vice versa.

During the main block of each task, the response buttons were already displayed before the stimulus appeared so that information on the required reaching distance could potentially influence the perceptual decision. We also included a control block for each task, during which the reaching distance of the response choice became only visible once the decision was made. We did so by presenting the response buttons after participants had already started reaching in the direction of their choice. This control block was used to check whether the bias towards the least costly option was indeed absent when we did not provide explicit information about movement costs. It establishes a baseline for comparison with the main block, as it reflects intrinsic biases—such as a preference for judging the left Gabor as oriented more upright in the absence of perceptual evidence—rather than biases induced by motor costs. For the orientation discrimination task, we also conducted a follow‐up experiment with a different group of participants, where we included a block with a third response button that participants could choose when they were uncertain of their choice. This served to examine whether the decision bias due to motor costs still holds when participants were not guessing but indeed perceived an orientation difference between stimuli. The methods and results of the follow‐up experiment are reported in Appendix S1.

The order of tasks and blocks within a task was counterbalanced across participants. Each of the four blocks consisted of 300 trials. This number was based on pilot experiments for which we were able to obtain reliable psychometric curves for the two experimental tasks. Participants did not receive any feedback on their performance. We varied both the position (left or right) of the closest response button and the stimulus level across trials. The two response buttons were presented in one of the two configurations: either the left closer and the right farther or the right closer and the left farther. Therefore, we had two conditions: left closer and right closer. Stimulus level refers to the signed amount of perceptual evidence, that is, for the motion discrimination task, we varied the level from leftward coherent movement (negative stimulus levels) via no coherent motion to rightward coherent motion (positive stimulus levels), while for the orientation discrimination task, we varied the level from a clearly more vertically oriented Gabor wavelet on the left (negative stimulus levels) via no orientation difference to a clearly more vertically oriented Gabor wavelet on the right (positive stimulus levels).

We varied the stimulus level across presentations using staircases. For each of the two conditions, we used two staircases: one starting at a negative stimulus level, and one starting at a positive stimulus level. Per block, this resulted in four staircases that were interleaved. Within each staircase, the stimulus level at each trial was adjusted relative to the previous level by selecting stimulus parameters based on the participant's previous response: If the answer was ‘left’, the next level would contain more perceptual evidence towards the right choice, and vice versa. Staircases were interchanged randomly throughout the block but the stimulus level at each trial was contingent on the participant's prior response for that particular staircase. Staircases were reset at the beginning of each block. For both tasks, each staircase consisted of 75 trials, hence resulting in 300 trials per block.

**FIGURE 1:**
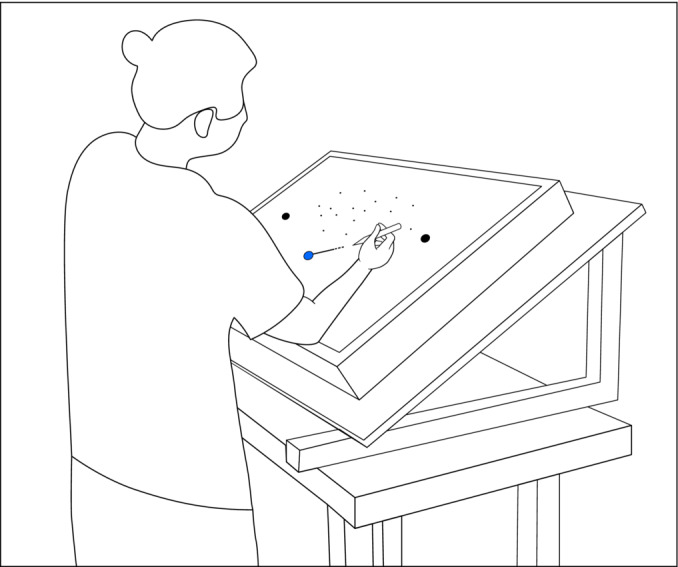
Experimental set‐up. Visual stimuli were presented on a tilted touchscreen. The participant responds by sliding a stylus from the blue start button towards one of the two black response buttons.

**FIGURE 2:**
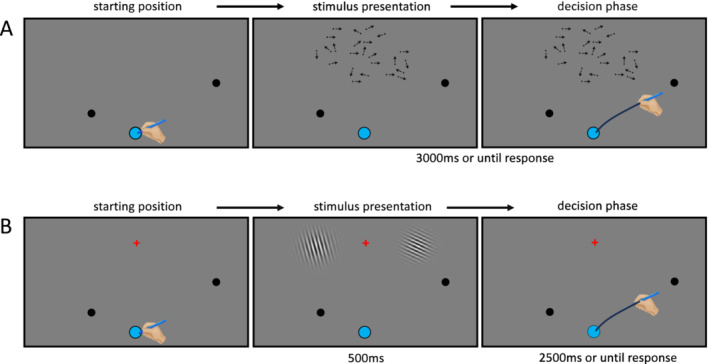
Trial sequence for the main block of both tasks. Every trial started with the presentation of the blue start button and the two black response buttons. (A) Motion discrimination task. Once participants tapped the start button, the random dot motion stimulus appeared and remained on the screen for 3000 ms or until a response was registered. The arrows indicate (fictive) dot motion directions for illustration purposes and were not visible during the experiment. Participants were instructed to slide the stylus from the start button to either of the two response buttons in accordance with the perceived rightward or leftward motion direction of the dot pattern. (B) Orientation discrimination task. Once participants tapped the start button, the Gabors flashed for 500 ms, and participants had 2500 ms to respond. Participants were instructed to slide the stylus from the start button to either of the two response buttons depending on the position of the more upright oriented Gabor. In this example, the left response button is closer, and the orientation difference is −50°, meaning that the right Gabor is set at 65° and the left one at 15°.

Both experimental tasks were created using PsychoPy 2022.2.5 (Peirce et al. 2019) and were presented on a 42‐in. IIYAMA PROLITE TF4237MSC‐B1AG touchscreen monitor with a 60‐Hz refresh rate, and a resolution of 1920 × 1080 pixels. The monitor was tilted in a 43° angle relative to horizontal and was positioned in landscape orientation. Participants were standing in a dimly lit room at a viewing distance of about 70 cm from the centre of the monitor and used a stylus to indicate their choices on the screen (see Figure 1). For both tasks, stimuli were presented against a grey background, and the starting point was indicated by a blue button at the lower middle part of the screen. The blue start button had a radius of 2.5 cm and was displayed at the monitor position (0, 7.5)—in centimetre relative to the body midline and the bottom of the screen. For the motion discrimination task (details in Section 2.2.1), the dots were presented in a cloud of 8.5‐cm radius centred at (0, 34). For the orientation discrimination task (details in Section 2.2.2), a fixation cross with a radius of 1 cm was presented at (0, 35). The Gabors were presented on the left (−15, 35) and the right (15, 35) side of the fixation cross and had a radius of 5 cm each. Additionally, two response options of 1.7‐cm radius were displayed as black circular buttons with a +35° and 145° angle movement trajectory from the starting point relative to the horizontal axis, respectively (see Figure 2). The required reaching distance between the blue start button and the closer response button was set at 15.4 cm and to the farther response button at 38.5 cm. For the left closer condition, the left button was presented at (−12.5, 16.5) and the right button at (31.25, 30), whereas for the right closer condition, the left button at (−31.25, 30) and the right button at (12.5, 16.5).

Before each block, there was a set of practice trials with feedback after every trial. The participants received 40 practice trials before the first block of a task and 20 practice trials before the second block of a task. During these practice trials, the two response buttons were presented at equal distances, either both at a close, left at (−12.5, 16.5) and right at (12.5, 16.5), or both at a far distance, left at (−31.25, 30) and right at (31.25, 30).

#### 2.2.1 Motion Discrimination Task

Random dot motion stimuli were created as movies of 3‐s duration using the Variable Coherence Random Dot Motion code for visual experiments (https://shadlenlab.columbia.edu/resources/VCRDM.html) and the Psychophysics Toolbox (PTB version 3.0.14) for Matlab. The movies featured 102 black dots that were distributed across three sets of 34 dots that were displayed in subsequent video frames in an alternating fashion. Dots had a diameter of 3 mm and were moving at a speed of 16.5 cm/s within a circular aperture of 24.6 cm with a transparent background. The starting positions of the dots were randomly chosen. The position of each dot was randomly reallocated with a probability of 1/6 per frame (i.e., the expected duration on screen was 100 ms) or once the dot disappeared beyond the visible range. For both movement directions, we created movies for coherence levels ranging from 0% to 100% with a step size of 5% (the percentage of dots moving in the same direction). Remaining dots moved in a pseudorandom direction: We chose random directions for half of the dots, and right–left mirrored these directions for the other half, to ensure that there was no residual horizontal motion component due to chance. For each coherence level, 15 unique movies were generated such that there was variation in the stimuli, and one was selected randomly for presentation in a trial. Leftward moving patterns will be referred to with negative values for the coherence.

For the main block of the motion discrimination task, participants were presented with the blue start button and the two black response buttons at the start of each trial. Once they tapped the start button, the moving dots stimulus appeared on the screen and remained visible for 3000 ms or until participants touched the response button with the stylus. Participants were informed that a subset of dots were moving towards the right or towards the left while the rest of the dots were moving in random directions. They were instructed to respond as fast as possible whether they perceived a pattern of movement towards the right or the left and to indicate their choice by sliding the stylus from the start button to the corresponding right or left response button. Afterwards, the start button could be tapped again to start the next trial. For the control block, the procedure was the same as for the main block, except that only the start button was presented at the beginning of a trial and the response buttons appeared at the moment that the stylus moved outside the start button, hence only after participants started moving to indicate their response. Participants were instructed to slide the stylus towards the side of the perceived dot motion direction without knowing the distance to move. The distance would become clear once the stylus had left the start button and the response buttons appeared.

As mentioned before, the percentage of coherently moving dots was varied using four staircases. Two staircases (one for each condition) started at 50% coherence and, the other two staircases started at −50%. On every subsequent trial, the coherence level was adjusted with a step size of 5% opposite to the direction of the participant's prior response. In other words, if the staircase was at −50%, a ‘left’ response would lead the next trial for this staircase to be at −45%. If the participant responded ‘right’, then the next trial for this staircase would be at −55%. The coherence limits were set at −100% and 100% but were never reached. In practice, coherence levels during the experiment ranged from −75% to 60%.

#### 2.2.2 Orientation Discrimination Task

The Gabor wavelet stimuli were generated using Python within Jupyter Notebook 7 (Kluyver et al. 2016), utilizing a Gabor generator implementation adapted from Mathôt's repository (Gabor‐patch‐generator, GitHub, https://github.com/smathot/gabor‐patch‐generator/tree/master/src). Specifically, the Gabors were generated using a Gaussian envelope (SD = 0.581 cm), the frequency was set at 4.13 cycles/cm and phase at 0.0 cycles, with a transparent background RGB at (−1,−1,−1), and Gabor pattern RGB at (255,255,255). Gabors were created for orientations ranging from 5° to 85° with respect to the vertical axis (all oriented from upper left to lower right) with a step size of 1°. The angles of the Gabors for each trial were symmetrical around 45°, and the orientation difference was defined as positive if the right Gabor was oriented more vertically. For example, if a trial would feature a 30° orientation difference, then the left Gabor would be oriented at 60° and the right one at 30°.

For the main block of the orientation discrimination task, participants were presented with the blue start button, the two black response buttons and a red fixation cross at the upper middle part of the screen at the start of each trial. They were instructed to fixate their eye gaze on the red cross and to tap the start button for the Gabor patches to appear. Once they tapped the button, the Gabor patches appeared on the left and right sides of the red cross for a period of 500 ms. Participants' task was to identify which of the Gabors was oriented more upright and to slide the stylus towards the response button at that side. For example, if the lines of the right Gabor were oriented more vertically compared to the lines of the left one, then participants had to slide the stylus from the start button until they reached the right response button. They were instructed to respond as fast as possible within a 3000‐ms time window. The next trial began once they made their response or 3000 ms after the Gabors appeared. For the control block, the procedure was the same as for the main block, except that every trial started with only the blue start button and the red fixation cross, and the response buttons appeared the moment that the stylus position first appeared outside of the area of the start button. Again, participants were instructed to start sliding the stylus towards the side of the most upright Gabor without knowing the distance to move. The distance would become clear once the stylus had left the start button and the response buttons appeared.

The orientation difference between the two Gabors was varied using four staircases. Two staircases (one for each condition) started at a 40° angle orientation difference and the other two started with a −40° difference. With each successive trial, the orientation difference between the Gabors was adjusted with a step size of 2° opposite to the direction of the participant's prior response. For example, considering a −40° difference between the Gabors, if the participant responded ‘left’, then the next trial for that staircase would consist of a −38° difference. If the participant responded ‘right’, then the next trial would consist of a −42° difference. The orientation difference could range from −80° to 80°, but again, these boundary values were never reached. Instead, the presented range of orientations varied from −46° to 56°.

### 2.3 Data Analysis and Statistics

To obtain an overall impression of task performance, we computed participants' reaction time, movement time and initial velocity, and the percentage of trials with a change of mind. We defined reaction time as the time from stimulus presentation until the moment that the stylus position first moved outside of the area of the start button, therefore indicating that participants started their response. We first computed the median reaction time for each stimulus level, block and task per participant to minimize the influence of a skewed data distribution. Then, we computed the mean of these medians across participants. We also computed the median reaction time per participant across all trials from all stimulus levels separately for each block and task and averaged these medians across participants. Furthermore, we calculated participants' movement time, which we defined as the period from the moment that the stylus position first moved outside of the area of the start button (i.e., reaction time), until the stylus position was inside the area of one of the response buttons, and hence, a response was registered. We computed the median movement time per participant across both tasks and blocks for the closer and the farther response option separately and subsequently averaged those medians across participants. We also computed the average of participants' median movement time across all trials for each task and block separately. Additionally, we computed the instantaneous (initial) velocity when the stylus first moved outside the start button based on the distance between its last recorded position inside and its first position outside. Again, for each participant, we took the median of these values per task and block separately and reported the group average.

To examine the presence of changes of mind while participants executed their movement, we analysed the sliding trajectories and calculated the percentage of trials in which participants started sliding in one direction but with the movement trajectory ending at the other response button. Participants' sliding trajectories can be traced through stylus position screen coordinates that were saved during the touchscreen contact. Based on the screen coordinates, we recreated the sliding trajectories, and we defined the initial direction based on the stylus position relative to the start button at the moment when the stylus position first appeared outside the area of the start button. In other words, if the stylus position first appeared on the right side of the start button, then the initial trajectory was assumed to be directed towards the right response button. If the stylus position then ended up inside the left response button and, hence, a left response was made, we defined this trajectory as a change of mind. This was determined for the main and control block separately to investigate whether information on the motor costs of the already made decision would change this decision. In other words, our aim was to determine whether the appearance of the response buttons after participants had already started indicating their choice in a control block would prompt them to switch their choice and opt for the closer button after observing the button positions. The trials with changes of mind were included in the analysis.

Next, for each participant and task, we fitted psychometric curves using psignifit version 3.0, a free toolbox for psychometric function estimation that relates response probability to stimulus intensity (Fründ, Haenel, and Wichmann 2011; Wichmann and Hill 2001). For each block type (main and control), we fitted two psychometric curves (one for each condition) to model the percentage of rightward choices based on the stimulus level, with each psychometric curve generated using data from two staircases. Psychometric curves were fitted as cumulative Gaussians by weighting all individual responses equally.

**FIGURE 3:**
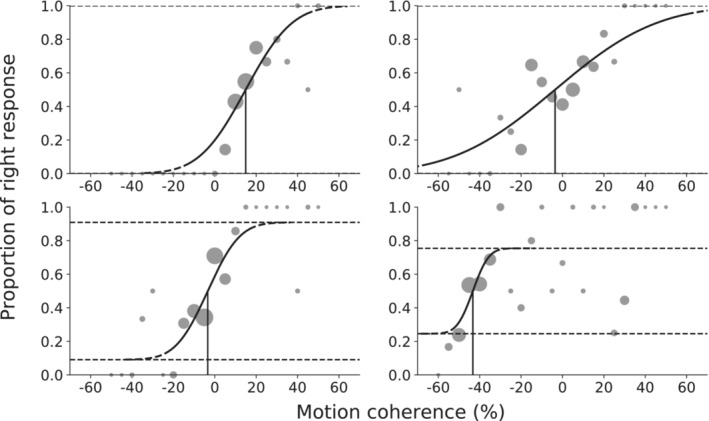
Example of psychometric curve fits for four different participants during the main block of the motion discrimination task. Each psychometric curve in this figure was fitted to data collected from two staircases (starting with leftward and rightward motion). The vertical line represents the fitted threshold, where 50% of responses were made to the right response button. The dots represent the mean percentage of rightward choice for each stimulus level, with the diameter of the dots proportional to the square root of the number of trials for each stimulus level. The points where the solid curve becomes dashed indicate the width, and the horizontal dashed lines indicate the lapse rate. Negative motion coherence levels correspond to perceptual evidence for leftward dot motion, positive motion coherence levels to rightward dot motion. The upper left and right panel show psychometric curves that were below the lapse criterion of 0.05, with thresholds 14.9% and −3.6% respectively, widths 58% and 127%, and lapse rates 1.06.10^−13^ and 1.27.10^−2^. The two lower psychometric curves are examples of curves that did not meet the lapse criterion. After visual inspection, the participant with the lower left psychometric curve, with a threshold of −3.4%, width 40% and lapse rate 0.09, was included in the analysis, whereas the participant with the bottom right psychometric curve, with a threshold of −43.3%, width 38% and lapse rate 0.24, was excluded.

By fitting the psychometric curves, we obtained three parameters: the *threshold*, representing the stimulus level where 50% of the choices were made to the right; the *width*, representing the difference between the 95% and the 5% point of the unscaled sigmoid; and the *guess/lapse rate* (a single value because we fitted for equal distances of the lower and upper asymptote from 0 and 1, respectively), along with the 95% confidence intervals (upper and lower boundaries) for each of the three parameters. An example of a psychometric curve fit is shown in Figure 3. We used the estimated lapse rate values of the individual psychometric curves to exclude participants with poor performance. The lapse rate represents the rate at which participants make stimulus‐independent responses. In case the lapse rate for one of the individual psychometric curves was higher than 0.05 (5%, equalling > 7 trials), we discussed with all authors whether to include the participant in further analyses or not. The decision was based on whether the fitted psychometric curve corresponded to serious task performance by the participant (see Figure 3). We identified seven participants in the motion discrimination task and five in the orientation discrimination task based on the lapse rate criterion, and we decided to exclude one person from the analysis of the motion discrimination task who was also the only participant to have lapse rates above 0.05 for multiple psychometric curves.

To compare participants' bias in decision making across blocks, we used the threshold value of each psychometric curve. Specifically, for each participant, we calculated the *decision bias* as the difference between the threshold value of the psychometric curve obtained from trials with the right response button closer and the threshold value of the psychometric curve obtained from trials with the left response button closer. This was done independently for the main and the control block, and separately for each task. Additionally, we calculated the 95% confidence intervals of the decision bias by taking the square root of the sum of the squared differences between each threshold and its corresponding upper/lower boundary of the 95% confidence intervals of the underlying threshold values. Then, we performed a Wilcoxon signed‐rank test to test whether the decision bias in the main and control block differed from zero. Additionally, we used a Wilcoxon signed‐rank for matching samples to compare the bias in the main and control. Finally, we examined whether the decision bias in the main block was correlated across tasks, that is, whether individuals with a larger decision bias in the motion discrimination task also showed a larger decision bias in the orientation discrimination task, by calculating the Spearman correlation coefficient between both decision biases across the 22 participants who performed both tasks. Statistical significance for all aforementioned tests was assessed using an alpha level of 0.05. Effect sizes for the Wilcoxon tests are reported as rank‐biserial correlations *r*_*rb*_. All analyses were performed using Python within Jupyter Notebooks 7 (Kluyver et al. 2016).

## 3 Results

We investigated whether explicit information of motor costs in terms of reaching distance can play a noticeable role in perceptual decision making by biasing choices towards the less effortful response option. Additionally, we investigated whether such a bias generalizes between perceptual tasks with different types of visual stimuli, in the form of a statistically significant response bias towards the least effortful option in both tasks. Twenty‐four participants performed two tasks: a motion discrimination task, identifying the overall direction of dot movement, and an orientation discrimination task, determining which of two Gabor wavelets was more vertical. For each task, the experiment included a main block, where response buttons were visible before the stimuli appeared, and a control block, where response buttons appeared only after a response was initiated. Each block consisted of 300 trials, with stimulus level adjusted using a staircase method based on participants' previous responses. Psychometric curves were fitted to model the percentage of rightward choices; the stimulus level for 50% rightward choices is referred to as the *threshold*. Decision bias was calculated as the difference in threshold values when the right or left response button was closer.

**FIGURE 4:**
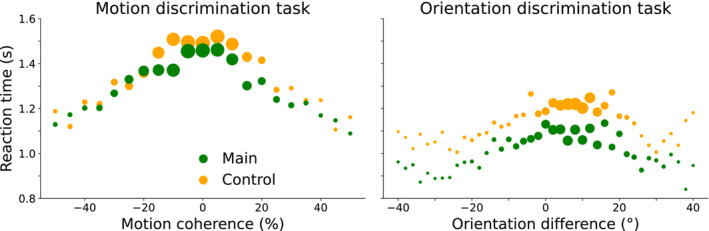
Reaction times as a function of stimulus level for each block, and task. Shown are the group‐average median reaction times per stimulus level in both tasks. The size of individual dots is proportional to the number of performed trials across all participants. Negative stimulus levels correspond to perceptual evidence for the left choice, positive stimulus levels to the right choice.

First, to gain insight into participants' overall performance on the two tasks, we examined their reaction times, movement times and initial velocity, and sliding trajectories. In Figure 4, median reaction times, averaged over all participants, are plotted against stimulus level for each block and task. As it appears, when fewer dots were moving coherently, and when the orientation difference between Gabors was smaller, participants took longer to initiate their response. Hence, reaction times tended to increase with the level of uncertainty in the task, as expected. In both tasks, there seems a small bias: the longest reaction times appear for a slightly negative motion coherence and a slightly positive orientation difference. Additionally, reaction times appear longer in the control blocks compared to the main blocks, and also during the motion discrimination compared to the orientation discrimination task. Specifically, across all trials from all stimulus levels, the group‐average median reaction time in the main block of the motion discrimination task was 1331 versus 1406 ms in the control block; and 1051 ms in the main block of the orientation discrimination task versus 1165 ms in the control block. The difference in reaction times between the main and control blocks was accompanied by a longer median movement time in the control blocks (256 ms for the main block of the motion discrimination task and 362 ms for the control block; 294 ms for main block of the orientation discrimination task and 439 ms for the control block). Median movement velocity upon moving the stylus outside the start button was lower for the control blocks (132 cm/s for the motion discrimination task and 89 cm/s for the orientation discrimination task) compared to the main blocks (161 cm/s for the motion discrimination task and 128 cm/s for the orientation discrimination task) suggesting that participants initiated their movement somewhat more cautiously in order to leave time to recalibrate their movement trajectory following the appearance of the target. As expected, participants' movement time to reach the closer response button was shorter (group‐average median across both tasks and blocks = 199 ms) compared to the farther response button (442 ms). When examining possible changes of mind based on participants' sliding trajectories, we observed that on average, participants changed their minds in about 1% of the trials. Specifically, during the motion discrimination task, participants changed their minds on average in two trials of the main block and in four trials of the control block, while during the orientation discrimination task, they changed their minds on average in three trials of the main block and in three trials of the control block.

**FIGURE 5:**
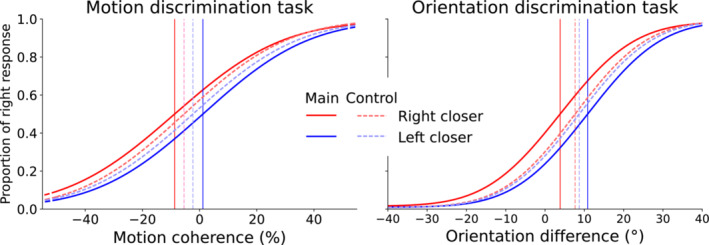
Average psychometric curves for each block, condition and task. The curves are based on the fit parameters averaged across participants. The vertical lines indicate the threshold values for the plotted curves. Negative stimulus levels correspond to perceptual evidence for the left choice, positive stimulus levels to the right choice.

**FIGURE 6:**
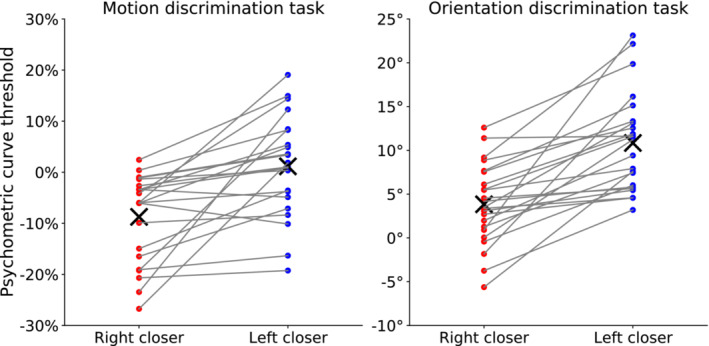
Fitted thresholds in the main block for each condition and task. Each data point represents the threshold value for a participant in the two tasks (panels) for the two conditions (colour within a panel); their mean values are indicated by an X. Grey lines connect the individual thresholds for the two conditions within a task.

To examine whether the difference in reaching distance to the response buttons induced a decision bias, we fitted psychometric curves for each condition. We found a clear effect for both tasks, as illustrated by the difference between the red (right response button closer) and blue (left response button closer) curves in Figure 5. The psychometric curves in this figure were plotted using the average fit parameters obtained across participants, for each condition, block and task separately. Descriptives of these parameters can be found in Table 1. A threshold near stimulus level 0 indicates that participants are equally likely to respond with a right or left choice when there is no perceptual evidence, hence indicating unbiased choices, while deviations from 0 indicate that more perceptual evidence towards that direction is needed to respond equally likely with a right or left choice. For the motion discrimination task, we observed that when the right response button was closer, participants needed on average more perceptual evidence in favour of leftward movement of the dots to respond with a left choice compared to unbiased choice. Nevertheless, when the left response button was closer, participants did not seem to need more perceptual evidence towards the right to respond with a right choice, that is, the curve resembled an unbiased choice. For the orientation discrimination task, participants showed a general bias towards the left choice, as they needed more perceptual evidence in favour of the right Gabor being oriented more upright compared to unbiased choice, both when the left response button was closer and when the right response button was closer. Individual threshold values for each condition in the main block of the two tasks are shown in Figure 6.

**TABLE 1:** Fitted parameters of the psychometric curves for each block, condition and task. Mean values across participants are listed with corresponding standard deviations in brackets.

	Motion discrimination task			Orientation discrimination task		
	Threshold (%)	Width (%)	Lapse rate	Threshold (°)	Width (°)	Lapse rate
Main block—right closer	−8.7 (8.6)	98 (41)	0.012 (0.028)	3.8 (4.5)	49 (29)	0.015 (0.046)
Main block—left closer	1.1 (9.8)	96 (42)	0.009 (0.031)	10.8 (5.3)	49 (28)	0.010 (0.019)
Control block—right closer	−5.4 (7.4)	95 (44)	0.004 (0.012)	7.6 (4.5)	47 (37)	0.008 (0.020)
Control block—left closer	−2.3 (7.3)	95 (46)	0.009 (0.024)	8.7 (3.4)	48 (40)	0.007 (0.022)

**FIGURE 7:**
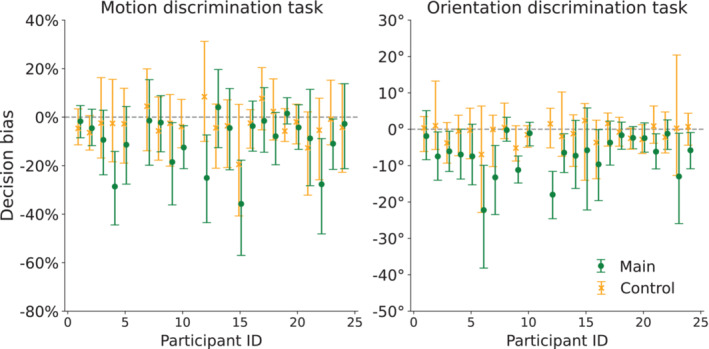
Individual decision bias for each block, condition and task. Each data point represents the decision bias for a single participant in the motion discrimination task (left panel) and the orientation discrimination task (right panel). Error bars indicate the 95% confidence intervals. A horizontal dashed line is drawn at 0 as a reference for the point of no decision bias due to reaching distance.

Our main dependent variable is the decision bias, defined as the difference between the threshold values across conditions. In particular, participants' decision bias is the difference between the threshold value for trials with the right response button closer and the threshold value for trials with the left response button closer. Individual decision bias values for each block and task are shown in Figure 7. For the motion discrimination task, the mean decision bias in the main block was −9.9% (95% CI = [−14.7, −5.1]), while the mean decision bias in the control block was −3.1% (95% CI = [−5.8, −0.5]). For the orientation discrimination task, the mean decision bias in the main block was −7.0° (95% CI = [−9.4, −4.6]), while the mean decision bias in the control block was −1.0° (95% CI = [−2.0, −0.1]).

In order to examine whether reaching distance had an effect on participants' choices, we examined whether the decision bias in the main block differed from zero. For the motion discrimination task, the Wilcoxon signed‐rank test revealed that participants' decision bias was significantly different from zero (Mdn = −6.2, *W* = 10, *r*_*rb*_ = −0.92, *p* < 0.001). The sign of the decision bias across participants signifies that the psychometric curve for trials with the right response button closer is shifted towards the left compared to the psychometric curve for trials with the left response button closer, meaning that participants needed a greater percentage of dot coherence movement towards the left to choose the farther left button compared to when the left button was closer. Or to phrase it for the other direction, participants needed a greater percentage of dot coherence movement towards the right to choose the farther right button compared to when the right button was closer. As can be seen in Figure 6, this effect was observed in 20 out of the 22 participants, and it is also evident from the average psychometric curves in Figure 5. Another way of looking at the decision bias is that, given a certain stimulus level, participants more often responded with a right response when the right response button was closer compared to when the left response button was closer (upward/downward shift of the responses in Figure 5).

Similarly, for the orientation discrimination task, a Wilcoxon signed‐rank test revealed that participants' decision bias was significantly different from zero (Mdn = −6.1, *W* = 0, *r*_*rb*_ = −1, *p* < 0.001). Again, the sign of the decision bias across participants signifies that the psychometric curve for trials with the right response button closer is shifted towards the left compared to the psychometric curve for trials with the left response button closer, indicating a response bias towards the closer response option. This effect was present in 23 out of 23 participants (see Figure 6), and it can also be seen from the average psychometric curves in Figure 5.

Surprisingly, a small but significant decision bias effect was also present in the control block of the motion discrimination task, where the reaching distance to the response buttons was only presented after participants had already initiated their response. The Wilcoxon signed‐rank test revealed that participants' decision bias was significantly different from zero (Mdn = −3.2, *W* = 55, *r*_*rb*_ = −0.56, *p* < 0.019). Therefore, we compared the decision bias between blocks to examine whether the motor cost effect in perceptual decision making was significantly larger in the main block than the control block. A Wilcoxon signed‐rank test revealed a significant difference between the decision bias in the two blocks (*W* = 51, *r*_*rb*_ = −0.59, *p* < 0.013). In particular, there was a significantly greater difference in the thresholds between conditions of the main block (Mdn = −6.2%) than between conditions of the control block (Mdn = −3.2%) (also visible in Figure 7). Specifically, the effect was larger for the main block compared to the control block for 15 out of 22 participants (see Figure 7).

On the other hand, the decision bias effect did not reach significance in the control block of the orientation discrimination task. In particular, a Wilcoxon signed‐rank test revealed that participants' decision bias was not significantly different from zero (Mdn = −0.4°, *W* = 81, *r*_*rb*_ = −0.41, *p* = 0.085). Again, we compared the decision bias between blocks. The Wilcoxon signed‐rank test revealed a significant difference between the decision bias in the two blocks (*W* = 12, *r*_*rb*_ = −0.91, *p* < 0.01). There was a significantly greater difference in the thresholds between conditions of the main block (Mdn = −6.1°) than between conditions of the control block (Mdn = −0.4°) (also visible in Figure 7). The effect was larger in the main block compared to the control block for 19 out of the 23 participants (see Figure 7).

**FIGURE 8:**
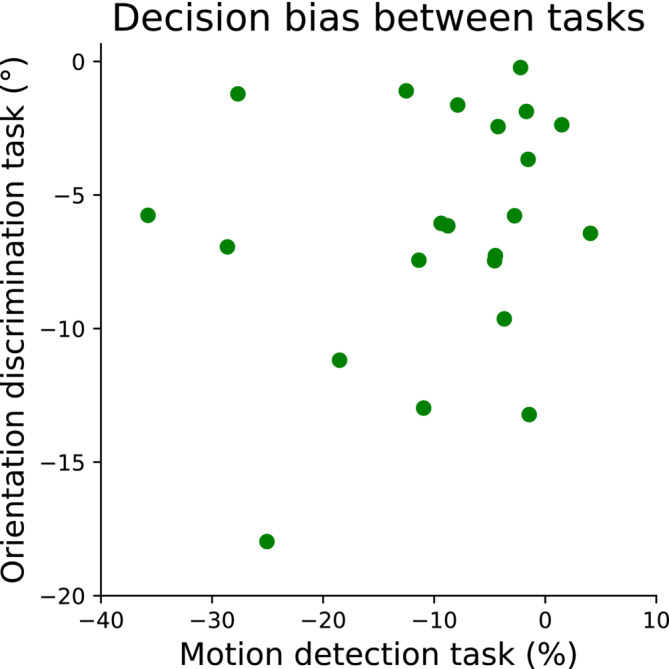
Individual decision bias in the main block of both tasks. Each data point represents the decision bias value for a single participant in the motion discrimination task (x‐axis) and the orientation discrimination task (y‐axis). No significant correlation was found between participants' decision bias in the two tasks.

Finally, we examined whether participants' individual decision bias was significantly correlated between the two tasks. A Spearman correlation revealed that this was not the case (rho(20) = 0.13, *p* = 0.562) (see Figure 8).

## 4 Discussion

The aim of the present study was to explore whether motor costs, in terms of reaching distance to the response buttons, influences perceptual decision making, and whether the effect generalizes between dynamic and static visual stimuli. So far, research in this field has been inconclusive, and evidence might point towards a contextual influence of motor costs on perceptual judgements, where motor costs can influence decision making when unaware of motor cost differences but not with explicit awareness of these differences. According to our findings, explicit information on the asymmetry of motor costs associated with the response options can introduce a decision bias towards the least effortful action. We observed that, when response options were presented before the stimulus appeared, participants required greater perceptual evidence to choose the farther option compared to the closer one. This effect was present in both experimental tasks, but the size of participants' individual bias was not correlated between tasks. In other words, participants with a strong decision bias in the motion discrimination task did not necessarily show a strong decision bias in the orientation discrimination task. Consequently, our findings suggest that explicit information of reaching distance influences perceptual judgements across different types of discrimination tasks but not in an idiosyncratic way.

There is one puzzling result in our study: the small bias in the control block. In this block, information on the motor costs of the response choice became only available after participants had already started the movement to convey their decision. So, a possible bias in this block should not be associated with visible future motor costs. In other words, during this block, we did not expect a difference in choosing the right or left side between the two conditions (right or left response buttons closer) since reaching distance information was available only after the initiation of the response. As the staircases were chosen randomly for each trial, it is not possible to anticipate which response button would be at a closer distance at any subsequent trial. Nevertheless, we observed a small but significant bias towards the closer option in the control block of the motion discrimination task and a trend for the same bias in the control block of the orientation discrimination task. A possible explanation would be that participants changed their minds as soon as they saw the response buttons, but as we reported in the second paragraph of Section 3, changes of mind were rare in both blocks. Another interpretation is that this small bias is not a true effect but occurred due to chance. As can be seen in Figure 7, the 95% confidence intervals of the individual decision bias overlap with zero for every participant. Additionally, the bias in the main block was greater than in the control block. We therefore argue that this small bias is a random fluctuation and not a worrying aspect of the findings.

To our knowledge, a perceptual bias induced by explicit motor costs associated with reaching distance has not been reported previously. Thus far, the impact of motor costs, in terms of movement effort, on perceptual judgements has been demonstrated in studies focusing on the biomechanical aspects of arm movements (Marcos et al. 2015) and movement resistance (Hagura, Haggard, and Diedrichsen 2017). We extend this line of research showing that reaching distance as a different aspect of motor costs also affects perceptual judgements. Moreover, we provide evidence that it can do so even with explicit knowledge of motor costs, in contrast to Manzone and Welsh (2023) who suggested that explicit motor cost manipulations might not produce a bias in perceptual judgements. Additionally, Manzone and Welsh (2023) argued that the subtle and implicit manipulations of motor costs used in previous studies (Hagura, Haggard, and Diedrichsen 2017; Marcos et al. 2015) are not ecologically valid, since information about action effort is usually available in real life. Nevertheless, in our experiment, although information about action effort was available and explicit before the presentation of stimuli, there was still an effect on perceptual decisions. Moreover, the decision bias could be detected in almost all individual participants, demonstrating the consistency of this effect.

Our results are consistent with studies suggesting that information available at an early stage of the decision making could exert a strong influence over this process (Wang 2002). In the experiment of Manzone and Welsh (2023), the effort manipulation was made quite clear to the participants, as a resistance band was used to induce greater movement effort on one side. As the manipulation was consistent across trials, participants might have implicitly adapted to it. Their manipulation might also have allowed participants to become aware of the aims of the study and consciously prevent motor costs from influencing judgements as it would decrease their performance. In our study, although knowledge of reaching distance was explicit, action options were interchanged randomly between trials to produce a subtle experimental manipulation. Therefore, future research could explore whether explicitly instructing individuals to disregard motor costs, thereby preventing their impact on performance, can effectively reduce perceptual bias.

A strong feature of our design is that the inclusion of a right closer and left closer condition in our experiment allowed us to focus specifically on the bias induced by motor costs while controlling for several confounding factors. In particular, individuals might show preferences for reaching towards a particular side of the body, either because of individual biomechanical and physical factors, such as handedness, or because of habitual patterns. These potentially confounding factors were accounted for by comparing the thresholds of the psychometric curves fitted for trials with the right response button closer against thresholds of the psychometric curves fitted for trials with the left response button closer. In particular, by examining the difference in the thresholds, we focused on the curve shifts, which allows us to effectively investigate the change in the amount of perceptual evidence needed to choose an option when the movement effort associated with this option increases. Thus, even if participants showed a general bias towards one option (which seemed to be the case as can be seen from the predominantly negative offsets for motion discrimination and the mostly positive ones for orientation discrimination in Figure 6, corresponding to the longer reaction times in Figure 4), keeping all things constant, and manipulating only the movement effort associated with a choice, we observed that participants needed greater evidence to choose a response when it was at a farther distance compared to when it was closer.

Furthermore, we investigated participants' commitment to the initial response trajectory by examining the sliding trajectories in the main and control blocks. In other words, if trajectories reflected more changes of mind in the control condition, we would know that the presentation of information of motor costs after initiating action response would influence their original decision and perhaps make them switch their response to the least effortful one. However, sliding trajectories showed that participants rarely changed their mind in both blocks. This is in line with previous literature suggesting that changes of mind are rare when switching between choice targets requires a large energetic cost, such as making a large deviation from the initial movement direction, as in our experiment (Burk et al. 2014; Lepora and Pezzulo 2015). Additionally, changes of mind are sensitive to time constraints, with less frequent changes of mind when there is less time to respond (Lepora and Pezzulo 2015). Neurophysiological evidence suggests that individuals need at least 200 ms of evidence accumulation to reach a decision and 200 ms to initiate a behavioural response, while there is a refractory period of 400 ms after the decision initiation during which new sensory information cannot influence the initial choice (Burk et al. 2014). In our experiment, participants took on average approximately 199 ms to reach the closer response button and 442 ms to reach the farther one. This means that participants would likely already have reached the response button or were too close to it for a change of mind to be beneficial, assuming motor cost considerations were driving their decisions. Taken together, it is unlikely that changes of mind occurred in response to additional information about the least effortful option.

An interesting aspect of our findings is the generalizability of motor cost effects to a task not studied before in the context of embodied perceptual decision making. In particular, most research on the influence of action on perceptual decisions employed the random dot motion paradigm (Burk et al. 2014; Hagura, Haggard, and Diedrichsen 2017; Manzone and Welsh 2023; Marcos et al. 2015). In our study, we examined the influence of motor costs on a different discrimination task employing static stimuli, namely, an orientation discrimination task using Gabor wavelets, which revealed the effect of motor costs on perceptual decision making to be generalizable. However, we found no evidence for the individual perceptual decision bias to be an idiosyncratic trait. It has previously been suggested that there are variations in participants' sensitivity to action effort (Burk et al. 2014; Saleri Lunazzi, Reynaud, and Thura 2021). This could mean that differences in how sensitive participants are to action effort may impact the extent to which effort is integrated into perceptuomotor decision making, and thus the extent by which motor costs influence perceptual judgements (Burk et al. 2014). Nevertheless, our findings did not provide support for this notion, which can be due to differences in task characteristics. In particular, the motion discrimination task involves making decisions based on accumulating evidence over time, which demands sustained attention, while the orientation discrimination task involves making rapid decisions based on a brief presentation of stimuli. Therefore, interindividual differences in tracking and interpreting changing stimuli over time and sensitivity to spatial orientation might influence participants' performance across these tasks and also influence the effect of motor costs on perceptual decision making. It is possible that the current sample size was not sufficient to detect remaining within‐subject consistencies across tasks. However, previous research with a larger sample size (*n* = 79) and a Bayesian statistical analysis also indicated a lack of generalization of individual embodied biases between walking and manual movements in a reward decision task (Grießbach et al. 2023). Future studies could explore in more detail whether individual susceptibility to decision bias due to motor costs is influenced by certain task‐specific perceptual or cognitive aspects and/or personality traits. Additionally, it is also worth examining potential differences in the effects of different types of motor costs on perceptual biases. For example, it has been suggested that temporal action costs, in terms of the duration that a response choice takes, can have a greater influence on decision making than energy costs, that is, motor costs (Saleri Lunazzi, Reynaud, and Thura 2021).

While this study provides evidence for the influence of motor costs on perceptual decision making, it does not give full insight into its underlying mechanisms. Since participants were forced to choose between left versus right responses, it is possible that the decision bias does not reflect an altered decision‐making process but is due to trials in which participants were uncertain about their decision and then chose the closest response button. We therefore conducted the follow‐up experiment presented in Appendix S1 where participants performed the regular main block of the orientation discrimination task and a block with a third ‘I don't know’ response button that participants could choose when they were uncertain of their choice. For both blocks, we found that reaching distance led to a decision bias towards the closer response option that significantly differed from zero, hence replicating the findings for the main block of the original experiments with a different group of participants. Moreover, in the follow‐up experiment, the decision bias did not significantly differ between the main and ‘I do not know’ block, showing that the findings of the original experiments are not simply due to a cost–benefit analysis in trials where participants experienced uncertainty. On the other hand, our results do not allow us to say whether the bias arises in the perceptual or decision‐making part as these are difficult to disentangle experimentally. The model‐based approach by Hagura, Haggard, and Diedrichsen (2017), however, showed that the manipulation of physical resistance in their study did not influence the accumulation rate of sensory evidence but biased the decision towards the direction of the least effortful motor response. This suggests that motor costs may act at the level of the decision phase rather than altering the sensory representation. We are also unable to strictly disentangle the influence of explicit versus implicit knowledge on the decision bias. In particular, while the nature of the influence is clear—explicit information of motor costs is associated with perceptual decision making—we cannot be certain whether it was explicit knowledge of motor costs that biased responses towards a least effortful option or implicit knowledge acting through the high repetitive task demands and lack of engagement of participants. Nevertheless, explicit information about motor costs was associated with perceptual decision making in our study, contrasting with other studies where implicit presentation of motor costs showed an association, while explicit information did not. Hence, future studies could focus on developing methodologies that can independently measure these influences, similar to what has been done for motor adaptation (Maresch, Mudrik, and Donchin 2021).

Further limitations of our study relate to the generalizability of the findings to the broader population and the study's ecological validity. Our sample consisted of university students, making it homogeneous in terms of age, cognitive abilities, educational background and socioeconomic status. As a result, the findings might not be generalizable to the broader population, making it essential to replicate the findings across diverse populations. Furthermore, although our research question aligns with real‐life scenarios where decisions are made in the context of their associated actions, the experimental set‐up lacks true ecological validity as the tasks do not reflect real‐world settings in terms of stimuli and action choices. Specifically, the tasks are abstract and simplified representations of perceptual tasks, utilizing stimuli not commonly encountered outside laboratory settings. In real‐world scenarios, motion and orientation discrimination often involve dynamic and multisensory environments, rather than sequences of visual stimuli presented on a screen. Finally, in real‐world scenarios, action choices and efforts associated with decisions are rarely as simplified and controlled as in our experimental design, since response mechanisms usually involve multifaceted factors and motor costs are influenced by numerous aspects beyond physical distance. Therefore, future studies should explore more naturalistic settings to better understand the implications of motor costs on decision making.

The results of this study have implications both for scientific theory and real‐life situations. More specifically, our findings provide evidence in line with ideas on embodied decision making, emphasizing the interplay between action and perception. Therefore, the motor system is not, as previously assumed (Donders 1969; Gold and Shadlen 2007; Oppenheimer and Kelso 2015), merely an effector of the decision, but the available action options play a significant role in how we perceive and make decisions due to our physical abilities and constraints (Cisek and Kalaska 2010; Lepora and Pezzulo 2015; Wispinski, Gallivan, and Chapman 2018). While this framework shift has great implications for research in psychology and neuroscience, real‐life applications might benefit from considering decision making from an embodied perspective as well. For example, in order to encourage healthier behaviours or environmentally friendly actions, interventions could be implemented that make desirable choices less effortful. In addition, understanding the impact of motor costs on perceptual decisions can lead to more user‐friendly designs for products and devices, including mobile apps, websites and gaming interfaces. Finally, rehabilitation interventions could potentially improve patient outcomes and adherence to treatment plans by taking into account movement effort.

## Author Contributions

**Eleonora E. Assarioti:** conceptualization, data curation, formal analysis, investigation, methodology, software, visualization, writing – original draft. **Robert J. van Beers:** conceptualization, methodology, supervision, writing – review and editing. **Jeroen B. J. Smeets:** conceptualization, methodology, supervision, writing – review and editing. **Bernadette C. M. van Wijk:** conceptualization, funding acquisition, methodology, supervision, writing – review and editing.

## Conflicts of Interest

The authors declare no conflicts of interest.

### Peer Review

The peer review history for this article is available at https://www.webofscience.com/api/gateway/wos/peer‐review/10.1111/ejn.70006.

## Supporting information


**Appendix S1.** Follow‐up experiment.

## Data Availability

Data and code are available on https://osf.io/kb3az.
