# Supplementary material for: Reaching Distance Influences Perceptual Decisions
Source: Eur J Neurosci. 2025 Feb 2;61(3):e70006. doi: 10.1111/ejn.70006 (PMC11788607; doi:10.1111/ejn.70006)
Supplement: Supplementary file 1 — Appendix S1. Follow‐up experiment. [file EJN-61-0-s001.pdf]

# Appendix 1. Follow-up experiment

## Introduction

We conducted a second experiment to gain more insight into the origin of the decision bias found in the first experiment. As participants were forced to choose between two options in our main experiment, the observed bias might not be due to a change in the decision-making process itself, but to trials in which participants were uncertain about their choice, and then chose for the nearest response option. To address this limitation, we conducted a follow-up experiment in which participants had the option not to choose the left or right response button if they were uncertain about their decision. This allowed us to examine whether motor costs would still influence perceptual choices when participants are confident about their decisions.

## Methods

We recruited a new sample of 22 university students (13 male, 9 female) from the same participant pool. The experiment was conducted for the task that yielded the strongest effect of motor costs, namely the orientation discrimination task. We kept the main block as originally designed but replaced the control block with a revised version. In the revised control block, participants saw all response buttons on the screen before stimulus presentation, as in the main block. The only difference was that there was a third "I don't know" button, which participants could select if they were unsure of the answer. The "I don't know" button was red and was positioned directly above the blue start button at the same distance as the closer option (see Figure A1). This adjustment allowed us to examine whether motor costs influence perceptual judgements when participants were not guessing but were confident about their response. We refer to the main block of this task as "Forced choice" and to the revised control block as "Free choice".

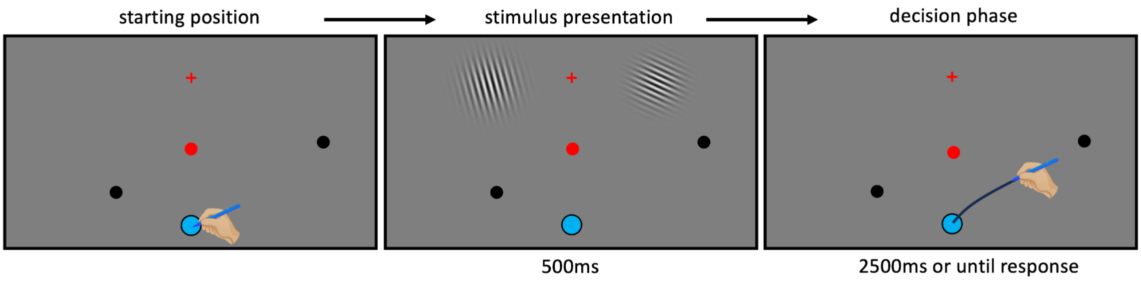


**Figure A1. Trial sequence for the Free choice block.** Compared to the main experiment (Forced choice block), a red "I don't know" button was additionally presented that participants could select in case they were uncertain about the orientation difference between the Gabors. See Figure 2 of the main text for further details of the trial sequence.

Similar to the main experiment, we included 4 staircases for both blocks, starting with a -40°/40° stimulus level and adjusting in the opposite direction of the previous response by a 2° step. When participants selected the "I don't know" option once, the stimulus level remained constant. After two or more consecutive "I don't know" responses in the same staircase, the

stimulus level was adjusted towards more perceptual evidence. For example, considering a -10° difference between the Gabors, if the participant responded “I don't know”, then the next trial for that staircase would feature a -10° difference, and if the participant responded “I don't know” again then the next trial would feature a -12° difference.

We fitted psychometric curves using the fraction of rightward responses for each stimulus level, i.e. using only trials where participants made a choice rather than responding “I don't know”, and obtained three parameters, the threshold, width and lapse rate. Similar to the main experiment, we visually inspected the psychometric curves that yielded a lapse rate higher than 0.05 and decided whether to exclude the participant. We decided to exclude three out of the six participants that had at least one psychometric curve meeting this criterion.

To compare participants' bias in decision making across blocks, we used the threshold value of each psychometric curve. Specifically, similar to the main experiment, we calculated the *decision bias* as the difference between the psychometric curve threshold values fitted with trials featuring the right response button closer and with trials featuring the left response button closer, independently for each block. We also calculated the 95% confidence intervals of the decision bias in the same way as for the main experiment. Finally, we used Wilcoxon signed-rank tests to examine whether the decision bias in the Forced choice and Free choice blocks differed from zero and from each other.

**Results**

On average, the “I don't know” button in the Free choice block was chosen during 43 trials (about 7% of the trials), with numbers varying from 1 to 105 for individual participants. The button was only chosen during trials with low perceptual evidence (in more than 90% of cases it was chosen for an absolute orientation difference below 16°). We infer from this that participants indeed used this button to indicate the lack of a confident choice about the orientation difference between the Gabors.

In order to examine whether reaching distance had an effect on participants' choices, we examined whether the decision bias in the Forced choice and Free choice blocks differed from zero. A Wilcoxon signed-rank test revealed that participants' decision bias was significantly different from zero for both the Forced choice block (Mdn = -2.94, $W = 3$, $r_{rb} = -0.96$, $p < 0.001$), and the Free choice block (Mdn = -3.48, $W = 13$, $r_{rb} = -0.86$, $p < 0.001$). The sign of the decision bias across participants signifies that the psychometric curve for trials with the right response button closer is shifted towards the left compared to the psychometric curve for trials with the left response button closer, indicating a response bias towards the closer response option for both blocks. Figure A2 illustrates that this effect was present in 18 out of 19 participants in the Forced choice block and in 17 out of 19 in the Free choice block. Finally, a Wilcoxon signed-rank test revealed that there was no significant difference between the decision bias in the two blocks ($W = 92$, $r_{rb} = -0.03$, $p = 0.921$).

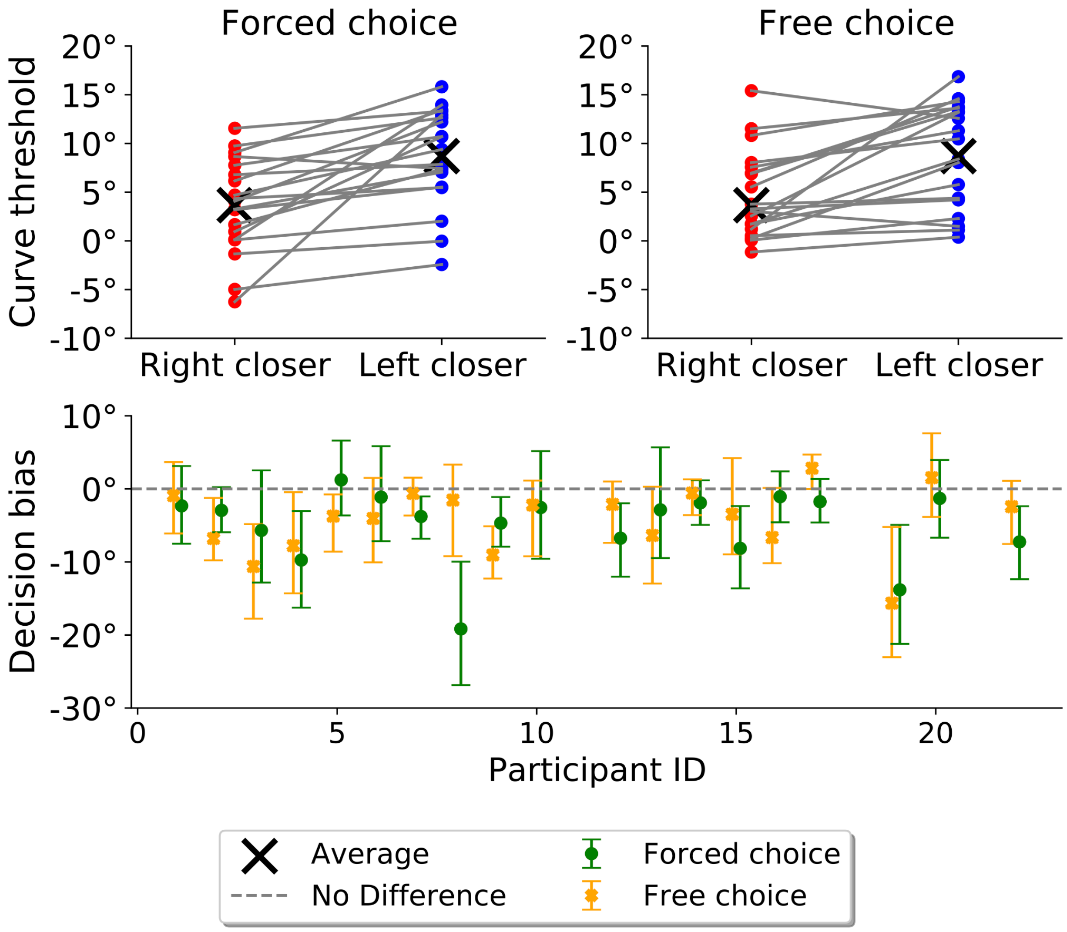


**Figure A2. Fitted thresholds and individual decision bias for the two blocks of the follow-up experiment.** The Forced choice block was identical to the main block of the main experiment. The Free choice block contained a third “I don’t know” button that participants could choose if they were uncertain about the orientation difference of the stimulus. In the upper two panels, each data point represents the threshold value for a participant in the two blocks (panels) for the two conditions (colour within a panel); their mean values are indicated by an X. Grey lines connect the individual thresholds for the two conditions within a task. In the bottom panel, each data point represents the decision bias for a single participant. Error bars indicate the 95% confidence intervals. A horizontal dashed line is drawn at 0 as a reference for the point of no decision bias due to reaching distance.

### Discussion

We replicated the finding of a significant decision bias when participants were forced to choose between left and right response options, as reported for the main experiment. Moreover, the bias was also significant and similar in size and direction for the Free choice block, in which we added a third button that participants could select if they were uncertain of the perceptual evidence. Despite (on average) frequent use of this button by the participants, the decision bias still remained. These results suggest that the decision bias does not arise from a simple cost-benefit analysis if participants are uncertain but that motor costs, in the form of reaching distance, play a genuine role in shaping perceptual decisions.
